# The Hospital Officer's Bookshelf

**Published:** 1912-06-01

**Authors:** 


### THE HOSPITAL OFFICER'S BOOKSHELF.

The Care of Infants and Young Children in Health. By Mildred M. Burgess, M.D. (Lond.). Second edition. (London: H. K. Lewis. 1912. Price 1s. net.)

We have so recently recorded our favourable opinion of this little book that we need do little more now than to draw attention to the fact that a second edition has already been called for. No great changes have been incorporated, but some extension has been allowed in the chapter on "Growth and Development." This very practical little book forms an admirable guide for mothers, teachers, and health visitors. It follows the syllabus of the infant-care course of the London County Council, and contains just the right sort of information set out in just the right sort of style. If we may hazard a criticism it is to suggest that a temperature of 90° Fahr. for the baby's bath errs on the side of coolness; at any rate, the water should be hotter than that at the commencement of operations, for the baby is not immersed in the bath until some minutes after the washing is begun.

The Bradshaw Lecture on Some Points in Heredity. By R. Clement Lucas, F.R.C.S. (Adlard and Sons. 1912. Price 2s.)

Mr. Lucas is a strenuous opponent of the "diathetic" school of clinicians, and in deriding the catchwords of a bygone generation he is easily able to make some very telling points. His assumption, however, that the old hypothesis is so totally demolished that none of its adherents dare lift his voice is not quite justified; for Sir Dyce Duckworth has lately written in defence of the diathetic pathology. Still, it may be freely granted that modern scientists are much more in agreement with Mr. Lucas than with the diathetists. It is hardly worth while to analyse further the contentions which the famous Guy's surgeon puts forward, for the lecture is so pithy and so easily read that those who take an interest in it had much better study it in the original. It suffices to testify that half an hour devoted to the task will be well and agreeably spent, and that whoso will read and mark the author's observations and speculations is unlikely to suffer any inward indigestion therefrom.

Clinical Methods. By Robert Hutchison, M.D., F.R.C.P., and Barry Rainy, M.D. Fifth edition. (Cassell & Co. 1912. Pp. 656. Price 10s. 6d.)

This excellent text-book during the sixteen years since it was first published has been a familiar and valued friend to the British medical student. It is questionable whether any other book has been so universally recognised as indispensable to the budding clinician, especially in the stages of ward work as clinical clerk. Certainly none has been so popular and so widely circulated. Nor is it only the novice that depends on "Hutchison and Rainy" for help in the ever more complex processes of diagnosis; for general practitioners and specialists alike know that this is one of the few books which they cannot do without. In the present new edition there is much that is new, and it is evident that no pains have been spared to make and keep the text-book thoroughly up to date. In this endeavour, it is only necessary to say, the authors have succeeded as conspicuously as formerly; and their already lengthening innings is clearly destined to last for a very long time yet. Their book is the best we know of in this field, and it should be on the shelves of everybody who is practising medicine, whether in a hospital or elsewhere.

The Etiology, Diagnosis, and Prophylaxis of Pulmonary Tuberculosis. By Alfred Harris, M.B., D.P.H. (Bristol: J. Wright and Sons. 1912. Pp. 126. Price 2s. 6d. net.)

As a well-thought-out epitome in a reasonable compass of the facts, as at present ascertained and believed, concerning the natural history of the disease which is commonly called by the public consumption, this monograph is unexceptionable. But it is not easy to see quite for what section of the community it is intended. It is obviously not for the lay public; it is not of much use to the ordinary practitioner or the consultant; it is far too elementary for the use of sanatorium medical officers; and surely, too, public health officials are quite familiar with the material facts as given here. Nor is there any original contribution to knowledge in the book. Still, if those there be to whom such a compendium is likely to be useful, they may rest assured that Dr. Harris is a guide in whom they may trust.

Consumption: Treatment at Home and Rules for Living. By H. Warren Crowe, M.D. Oxon. Third edition. (Bristol: John Wright and Sons, Ltd. 1912. Price 1s.)

A book for practitioners to give their patients is somewhat of a novelty and the desirability of doing so may be open to doubt in most cases. As regards consumptives, however, an important part of their treatment consists in learning how to live, and the instruction necessary cannot be given without the expenditure of a considerable amount of time. Those who can get into a sanatorium will there learn some of the principles underlying the successful treatment of pulmonary tuberculosis, but for those who cannot live in a sanatorium this book provides a ready and straightforward didactic substitute. Dr. Crowe has written dogmatically and clearly, though, of course, the book is very limited. The main principles are certainly well put, and we can imagine that the observance of the rules laid down here would enable a consumptive to benefit himself very materially. Briefly, the book consists of rules on the subject of the relations between rest, exercise, and temperature, with explanations and reasons. We may quote Rule XII.: "When in doubt, rest," and also the advice to get wet rather than run for shelter. On the whole this little book is one which may be with advantage read by all sufferers from tuberculosis.

Lessons on Massage. By M. D. Palmer, a founder of, and examiner to, the Incorporated Society of Trained Masseuses. Fourth edition, pp. xvi. + 292. (London: Baillière, Tindall and Cox. Price 7s. 6d. net.)

Undoubtedly one of the most successful text-books on massage, Miss Margaret Palmer's compact work is now enjoying the distinction of a fourth edition. We have on previous occasions expressed a favourable opinion of this book, and we are glad to be able to say that the alterations and additions in this new issue are all on the side of improvement. Not only is the volume of value as an excellent teaching manual on the subject of massage, but it is also deserving of considerable praise as a text-book of anatomy. The illustrations are laudably clear, and there is a fair proportion of good coloured diagrams. The tables of muscles, giving their origins, insertions, nerve supplies, etc., should be particularly helpful to the student, who is also supplied with a list of the modern names of these and other structures in accordance with the Basle Convention. As regards massage proper, the author's teaching will be found to present the best modern methods in a thoroughly practical style. We can specially recommend this text-book for students preparing for the examinations of the Incorporated Society of Trained Masseuses.

British Pharmaceutical Codex 1911. Published by direction of the Council of the Pharmaceutical Society of Great Britain. (London: The Pharmaceutical Press, 72 Great Russell Street, W.C. 1911. Price 10s. 6d.)

The last issue of the Pharmaceutical Codex is dated 1907. Since that year a large amount of revision work has been done, and many old remedies have dropped out of favour, while others have had their claims acknowledged in a more scientific spirit than heretofore. But the codex, being the weighty pronouncement, in epitome, of the learned committee that sits on these claims, cannot deal lightly with the numerous varieties of new drugs and new remedies that compete for the practitioners' favour. We do not, therefore, find the official codex so interesting or so valuable a book as the annual résumés of new remedies issued by various continental firms of pharmaceutical chemists. But while it is comparatively silent with regard to new methods and new drugs, it contains a large amount of very useful, interesting, and here and there unconsciously amusing information. Among the "drugs" that have been included by the committee of experts we find "acidum uricum," which is our old friend of the physiological laboratory days when we attempted the murexid test. Uric acid, however, as the codex admits, "is said to be quite inactive" as a drug, Why then retain it in the codex? Another old friend is cuttlefish bone (Os sepiae), but its inclusion is perhaps warranted on the ground that some dentists are still in the habit of looking upon it as an indispensable ingredient in a tooth powder; while some medical missionaries apparently seem to think that it is a sovereign remedy in dysentery. It is, of course, nothing more than phosphate of lime, mixed with a very large amount of carbonate of lime, and about 10 per cent. of organic matter. The practitioner who has half an hour to spare would do well to devote that time of leisure to reading the rubrics "Action and Uses" of the various drugs enumerated in the codex. He will learn much from this perusal for, on the whole, once we have accepted the somewhat arbitrary manner in which the selection has been made, the summary of the physiological actions is fairly and clearly put. For instance, that on alcohol gives the case for and against this drug in a ten-line paragraph which we heartily wish the *Daily Mail* would induce its Medical Correspondent to foster for publicity in a quarter-column article. Again, the short notes on aloes, on sparteine, apocynum, digitalis, and the opium compounds are excellently informative. The codex is, as always, splendidly printed and bound.

A System of Surgery. Edited by C. C. Choyce, B.Sc., M.D., F.R.C.S. Pathological Editor, J. Martin Beattie, M.D., C.M. In three volumes. Vol. I. (Cassell & Co., Ltd. London: New York, Toronto, and Melbourne. 1911. Pp. 957. Price 21s. net.)

Many of us remember with pleasure the time when we first made acquaintance with Treves' "System of Surgery." This new edition of the work, under the editorship of the well-known Dean of the Greenwich Post-Graduate School, challenges comparison with its prototype. We cannot honestly make such a comparison until we have all the three volumes before us. Judging from the first volume only, which is concerned mainly with surgical pathology, we find the new system an admirable résumé of modern teaching. Surgeons are notoriously bad writers, and it is useless to look for the literary ability of a Wiseman or a Bilroth in modern essays, which are mainly compilations by men who very often are ignorant of the essentials of proper literary construction of a sentence. For this reason perhaps it is cavilling criticism merely to express the wish that Mr. Choyce—himself a writer of at least lucid and grammatical English—had sub-edited his contributors' essays with more severity. A few of them—we may instance the article on congenital syphilis, which is one of the most mediocre compilations that we have ever read in a work that lays claim to being of first-class merit—are so curiously inept in expression and turgid in construction that some of their sentences might usefully be cited as examples of how not to write scientific essays in English. This grumble apart—and we say again that Mr. Choyce is responsible for the fact that calls it forth only in so far that he has not used his blue pencil with greater freedom—the first volume is very commendable to all concerned. It makes a heroic attempt to be up-to-date, but necessarily fails as such a system must always fail. Its illustrations are for the most part excellent—some of the coloured ones remarkably fine in finish. The articles are for the most part by young men, and one misses the personal clinical experience that made certain of the essays in the older system so valuable. The editor's and Mr. G. Williams' article on "Wound and Wound Treatment" is one of the best in the book. Among other excellent and informative essays we may instance Mr. Cheatle's article on Suppuration, Mr. Nitch's on Gangrene (in which we note that nothing is said about tobacco gangrene), Dr. Keith's short essay on Drowning (wherein the Schafer method is given alongside the more efficient, less dangerous and far less barbarous Sylvester method), Mr. Raymond Johnson's admirable condensation on Tumours, Col. Lambkin's article on Acquired Syphilis, and Mr. Maynard Smith's contribution on Inflammation. The information given is sound and in consonance with the best teaching on the subject. Most of the articles conclude with a short and very incomplete bibliography. Full particulars are given of modern methods of clinical and pathological examination, and the descriptions are in general accurate and lucid. We congratulate Mr. Choyce on a work which is certainly a monument of energy and painstaking thoroughness. If the succeeding volumes are up to the technical standard of the first they will prove worthy successors of the old Treves. If they are a trifle more scholarly in diction they will have the added value of rivalling the literary excellence of their admirable prototype.

